# Clinically Malignant Breast Lesion in an Adolescent Girl: A Case Report

**DOI:** 10.3390/clinpract11030058

**Published:** 2021-07-02

**Authors:** Patrycja Sosnowska-Sienkiewicz, Przemysław Mańkowski

**Affiliations:** Department of Pediatric Surgery, Traumatology and Urology, Poznan University of Medical Sciences, Szpitalna Street 27/33, 60-572 Poznan, Poland; mankowskip@ump.edu.pl

**Keywords:** adolescent, antibiotic therapy, breast abscess, breast cancer, surgical treatment

## Abstract

Diseases of the breast in adolescent women are usually benign, and their treatment is simple using appropriate medical strategy and rarely surgical therapy. The whole team’s real challenge is when the girl presents malignant breast cancer symptoms such as a non-movable tumor, nipple discharge, nipple retraction, retraction of the skin, inflammatory infiltration of the breast, or ulceration. Presented here is a case of a 15-years-old girl with the features of a malignant neoplasm of the breast. There was an observed non-movable tumor, retraction of the nipple, inflammatory infiltration, and pain. The performed ultrasound and magnetic resonance imaging suggested a malignant lesion measuring 84 mm × 66 mm × 50 mm. After many diagnostic difficulties, the lesion evacuated spontaneously, and the abscess was diagnosed. In conclusion, not all features of a malignant breast tumor in adult women are typical for adolescent females. In young girls, breast diseases are usually benign, and appropriate diagnostics and therapy allow for an effective cure. Atypical breast lesions require the extraordinary cooperation of a multidisciplinary team.

## 1. Introduction

Diseases of the breast in adolescent women are usually benign, and their treatment merely uses appropriate medical strategy and rarely surgical therapy [1,2]. The most common lesions in this age group are fibroadenomas and breast growth disorders [3]. The whole team’s real challenge is when a girl presents malignant breast cancer symptoms such as a non-movable tumor, nipple discharge, nipple retraction, retraction of the skin, inflammatory infiltration of the breast, and ulceration [4].

We present a case of a 15-year-old girl with the features of a malignant neoplasm of the breast that made a big diagnostic challenge for the entire multidisciplinary team.

## 2. Case Report

**Figure 1:**
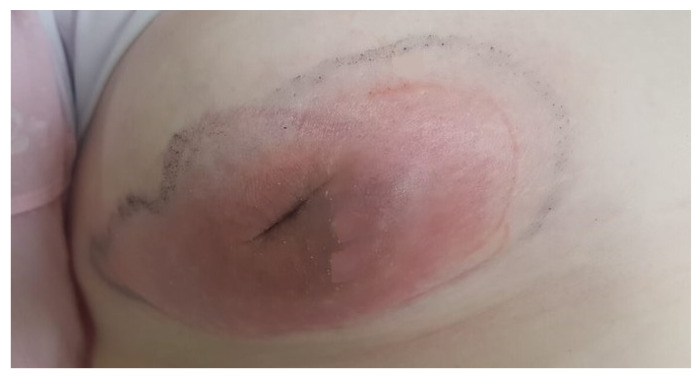
The clinical picture of the right breast tumor. Visible swelling, redness, and retraction of the right nipple.

A 15-year old girl was admitted to the Department of Pediatric Surgery, Traumatology, and Urology in Poznan. She came to the hospital with a disturbing lesion of the right breast. There was an observed non-movable tumor, retraction of the nipple, and inflammatory infiltration (Figure 1). The lesion was painful.

In blood laboratory tests, the only abnormalities were increased CRP (C Reactive Protein) levels (6 mg/L) and leukocytes (16 × 10^9^/L). Antibiotic therapy was started.

**Figure 2:**
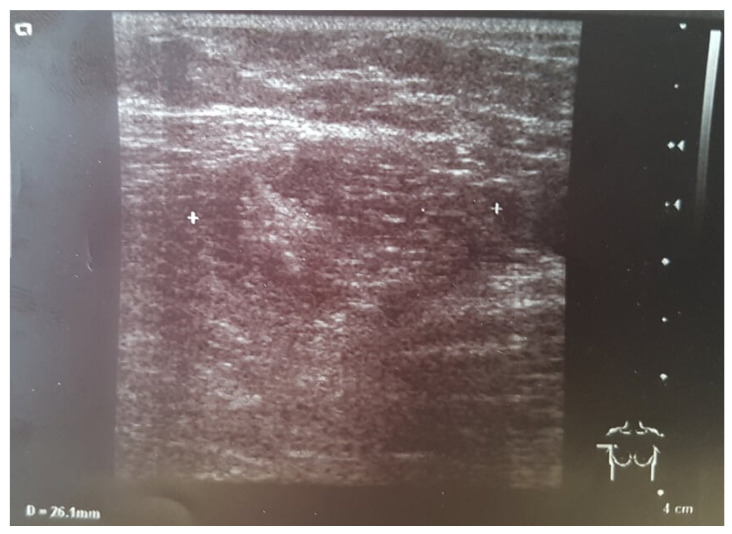
USG examination of the right breast. White characters indicate the range of the lesion.

During ultrasonography (USG) behind the right nipple, a thick-walled cyst with polycyclic contours measuring 25 mm × 14 mm was diagnosed. The lesion communicated with the dilated milk duct and the right retracted nipple. The left breast was normal. In both armpits, enlarged lymph nodes up to 20 mm were found (Figure 2).

Due to the clinical presentation, gynecological and oncological consultations were carried out immediately. A multidisciplinary team decided to control appropriate tumor markers, and perform magnetic resonance imaging (MRI) and fine-needle aspiration biopsy.

Tumor markers of breast cancer (CA 15.3, CEA) were negative.

**Figure 3:**
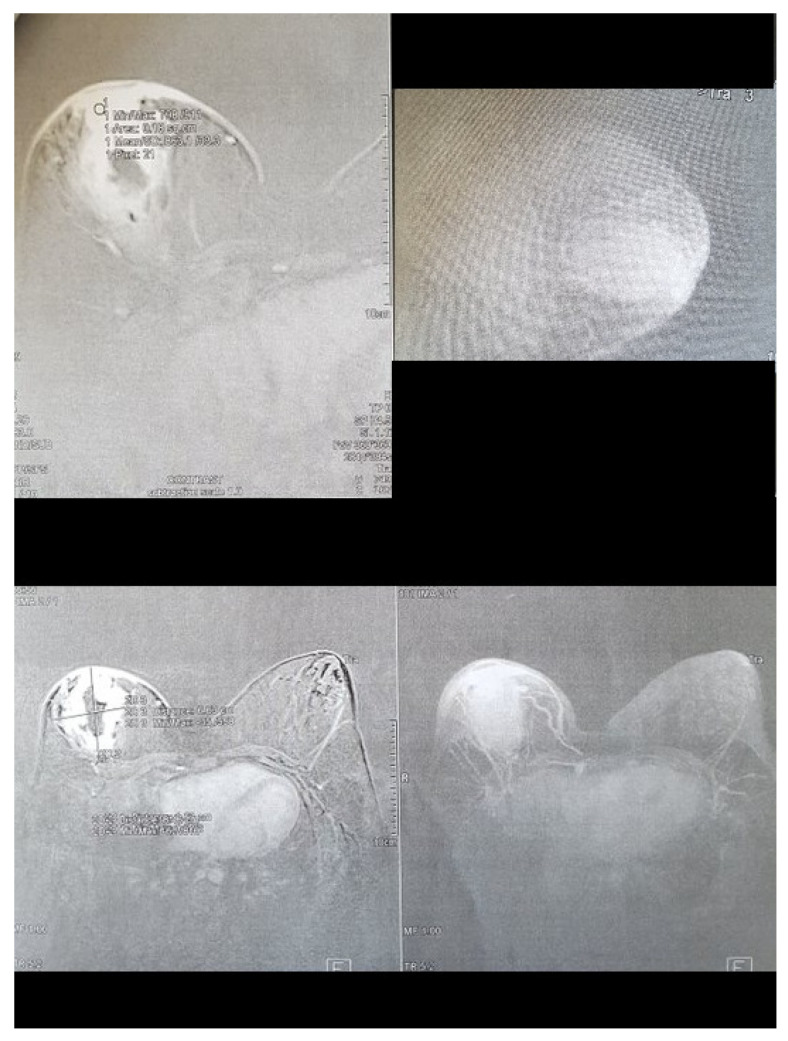
Magnetic resonance imaging of the right breast showing the following phases of the study. Visible contrast enhancement of the lesion, widening of the milk duct, and retracting the nipple.

In the MRI examination, a centrally located, irregular, poorly limited litho-cystic mass, which retracted the nipple, was diagnosed. There was an intense, marginal contrast enhancement of the lesion. The tumor was 84 mm × 66 mm × 50 mm and contained a centrally irregular fluid space of 48 mm × 20 mm. Swelling of the surrounding adipose and gland tissue was visible—its posterior pole infiltrated the pectoral muscle for a distance of about 16 mm. The areola’s skin and the central part of the breast were thickened to 8 mm and were intensely enhanced with contrast. Numerous abnormal lymph nodes were visible in both armpits. The radiology team suggested a malignant lesion (Figure 3).

**Figure 4:**
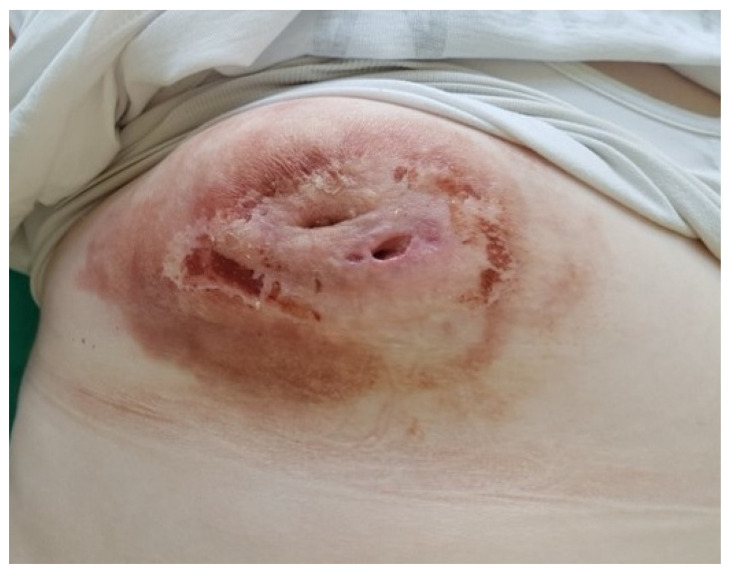
The right breast image at the end of therapy, after the evacuation of the purulent contents. Visible numerous fistulas and mild redness. Retraction of the nipple still present.

The broad-spectrum antibiotic therapy was not significantly effective. There were no clinical improvements and a significant decrease in inflammation markers. The effects of the applied therapy were low. The result of the fine-needle aspiration biopsy was undiagnosed, and another histopathological verification was necessary. Further diagnostic decisions were a considerable challenge for the entire team. The patient’s medical records were transferred to another center for consultation. During waiting for the consultation results, a significant amount of pus spontaneously evacuated from the right breast two weeks after the initial diagnostics and therapy. The material was collected for microbiological assessment. Methicillin-sensitive (MSSA) *Staphylococcus aureus* strains were isolated. Signs of inflammation with no cancer cells were described in the repeated cytological examination. The applied antibiotic was appropriate and continued. After the evacuation of the lesion, there was a spectacular clinical improvement (Figure 4).

Inflammatory markers normalized. A breast ultrasound showed a significantly reduced inflammatory infiltration of the breast.

After such a long time of diagnostics, therapy, and enormous challenges for the entire multidisciplinary team, a breast abscess was diagnosed.

## 3. Discussion

Various conditions can disrupt the normal development of breasts in an adolescent female. They lead to many types of pathologic lesions. The majority of these tend to be benign, but some of them can be unsettling to the patient, her family, and the treating physician.

A tumor, which seems fixed to the chest wall, with nipple discharge, inward inversion of the nipple, inflammatory infiltration of the breast, and ulceration are typical breast cancer symptoms—but in adult women. In adolescent women, they are possible, but not so typical. For example, the nipple retraction may or may not be a feature of the malignancy [4].

Pediatric breast cancer usually is characterized by the secretory variety and has less metastatic potential. However, cases of inflammatory and medullary breast cancers also were reported in girls and are more aggressive. In the case of symptoms presented by our patient, we could differentiate between breast abscesses and inflammatory breast cancer. Symptoms of the latter include: pain in the breast, skin changes in the breast area (pink or reddened areas often with the texture and thickness of an orange), a bruise on the breast that does not go away, sudden swelling of the breast, itching of the breast, nipple changes or discharge, and/or swelling of the lymph nodes under the arm or in the neck [5]. This similarity of symptoms prompted the treatment team to exercise such great therapeutic caution.

Surgical treatment for pediatric breast mass is complete resection while preserving normal breast development, when appropriate. Due to the small amount of data, surgical management of primary breast cancer in the pediatric patient remains controversial. Complete resection is always the main task during surgery; however, maintaining normal breast development should also be considered wherever possible. The necessity for axillary lymph node staging or axillary dissection remains unclear. Radiation and chemotherapy can be connected with an increased risk of further cancers in young patients; therefore, disadvantages and benefits should be carefully analyzed based on tumor type and stage of disease [4,5].

A very unclear diagnosis occurred in the case of our patient. The adolescent female presented alarming symptoms for the entire multidisciplinary team. The clinical condition of the patient suggested the malignant lesion’s features, but finally, the diagnosis was a breast abscess.

They can be distinguished as lactational and non-lactational or puerperal and nonpuerperal breast infections [6,7]. It is crucial to rule out more severe pathologies, such as breast cancer, when non-lactational females have breast abscess symptoms. Usually, diagnosis and therapy of typical abscesses are not difficult [6,7]. We know from the example of our adolescent patient that this is not the rule.

Nonlactating breast abscesses have a peak incidence in the fourth decade of life [8]. Our patient was 15 years old, which was also unsettling for us.

A correlation between breast abscess and diabetes and smoking was found [8]. It was not confirmed in our case.

The literature proved that obese females and African Americans have a greater incidence of breast abscesses [8]. The body mass index (BMI) of our patient was 31, indicating obesity.

The most crucial element of diagnostics, also in an abscess, is physical examination [6,7,8]. The differential diagnosis between benign or malignant breast mass, inflammatory breast cancer, and cellulitis should always be performed [4,7,8]. Leukocytosis and an increase in CRP levels can occur in any of the diseases mentioned above. In an ultrasound examination, abscesses can be observed as ill-defined masses with internal septations [7]. A breast cancer tumor is often seen as hypoechoic with irregular borders and may appear spiculated. Other ultrasound features suggesting breast cancer include non-parallel orientation (not parallel to the skin), a mass that is taller than wide, acoustic shadowing (a finding that indicates a solid mass), microlobulation (collections of small lobes), duct extension, a branching pattern, a mass within a cyst, and angular margins (an irregular or jagged appearance) [9]. Acoustic shadowing and angular margins were observed in our patient. The ultrasound specialist was also concerned about the solid element of the lesion coexisting with the cystic part.

Cancerous masses on MRI often present irregular or spiculated borders. Rim enhancement on the outside of the lesion is also common [9]. In our patient, an MRI showed an intense, marginal contrast enhancement, features of infiltration of surrounding tissues, and both cystic and solid components of the lesion.

The result of a fine-needle breast biopsy does not always give a definitive answer to the question about the lesion’s nature. In 20–30% of cases, the biopsy result is undiagnosed [6,7,8]. That was in our case, and it was decided to consult with experts outside our medical center. During this time, the lesion evacuated spontaneously, and this moment was a breakthrough in therapy.

The most common organism that caused a breast abscess is *Staphylococcus*, but in some cases, *Streptococci* and *Staphylococcus epidermidis* may also be involved [10]. In our case, after the evacuation of the lesion, methicillin-sensitive *Staphylococcus aureus* was diagnosed. There were no signs of a malignant tumor in the repeated cytological examination, only signs of inflammation.

Incision and drainage are the standards of treatment for breast abscesses. The patient may start therapy from antibiotics [7]. Until a spontaneous evacuation of pus, the antibiotic was not significantly effective in our patient.

In the case of signs of a malignant breast tumor, the patient should be referred to an appropriate specialized center [4].

## 4. Conclusions

When the breast lesion presents an atypical clinical course, it can cause significant distress to the patient, family, and doctors.

Ultrasound examination is the primary examination of breast changes in adolescent girls. However, in the case of diagnostic difficulties, an MRI is necessary.

Atypical breast lesions require the extraordinary cooperation of a multidisciplinary team, and in the case of signs of a malignant breast tumor, the patient should be referred to an appropriate specialized center.

## Data Availability

Data are available on request due to restrictions.
